# Quercetin inhibits macrophage polarization through the p‐38α/β signalling pathway and regulates OPG/RANKL balance in a mouse skull model

**DOI:** 10.1111/jcmm.14995

**Published:** 2020-02-13

**Authors:** Yu‐Wei Ge, Kai Feng, Xiao‐Liang Liu, Zhen‐An Zhu, Hong‐Fang Chen, Yong‐Yun Chang, Zhen‐Yu Sun, Hao‐Wei Wang, Jing‐Wei Zhang, De‐Gang Yu, Yuan‐Qing Mao

**Affiliations:** ^1^ Department of Orthopedic Surgery Shanghai Ninth People’s Hospital Shanghai Jiao Tong University School of Medicine Shangai China; ^2^ Department of Orthopedic Surgery Shanghai Jiao Tong University Affiliated Sixth People’s Hospital Shanghai China; ^3^ Department of 2nd Dental Center Shanghai Ninth People's Hospital College of Stomatology Shanghai Jiao Tong University School of Medicine Shanghai China; ^4^ National Clinical Research Center for Oral Diseases Shanghai China; ^5^ Shanghai Key Laboratory of Stomatology & Shanghai Research Institute of Stomatology Shanghai China

**Keywords:** macrophage, osteoclasts, polarization, signalling, titanium

## Abstract

Aseptic loosening caused by wear particles is a common complication after total hip arthroplasty. We investigated the effect of the quercetin on wear particle‐mediated macrophage polarization, inflammatory response and osteolysis. In vitro, we verified that Ti particles promoted the differentiation of RAW264.7 cells into M1 macrophages through p‐38α/β signalling pathway by using flow cytometry, immunofluorescence assay and small interfering p‐38α/β RNA. We used enzyme‐linked immunosorbent assays to confirm that the protein expression of M1 macrophages increased in the presence of Ti particles and that these pro‐inflammatory factors further regulated the imbalance of OPG/RANKL and promoted the differentiation of osteoclasts. However, this could be suppressed, and the protein expression of M2 macrophages was increased by the presence of the quercetin. In vivo, we revealed similar results in the mouse skull by μ‐CT, H&E staining, immunohistochemistry and immunofluorescence assay. We obtained samples from patients with osteolytic tissue. Immunofluorescence analysis indicated that most of the macrophages surrounding the wear particles were M1 macrophages and that pro‐inflammatory factors were released. Titanium particle‐mediated M1 macrophage polarization, which caused the release of pro‐inflammatory factors through the p‐38α/β signalling pathway, regulated OPG/RANKL balance. Macrophage polarization is expected to become a new clinical drug therapeutic target.

## 1 INTRODUCTION

Total joint replacement is a highly successful surgical technique that has been used clinically for decades. However, aseptic inflammation remains one of the most serious complications of total joint replacement.^1^ Aseptic inflammatory reactions further aggravate the formation of aseptic loosening around the prosthesis.^2^, ^3^ Once the aseptic looseness has formed, total joint replacement is recommended. Wear particles, such as titanium (Ti), ceramics and polymethyl methacrylate (PMMA), can cause this aseptic inflammation to be confirmed by the investigator.^3^, ^4^ Previous researchers have focused on the pathological mechanisms of osteoclasts formation.^5^, ^6^, ^7^, ^8^ However, few studies have shown the increased accumulation of macrophages at the beginning of wear particles. The macrophage cells are then further differentiated into osteoclasts with bone resorptive function.

Wear particles, which are generated at the interface of the implant and the bone, can exacerbate the aggregation of bone marrow macrophages (BMMs).^9^ Thus, they further promote the differentiation of BMMs into osteoclasts and the release of a large number of pro‐inflammatory factors such as interleukin‐1beta (IL‐1β), tumour necrosis factor alpha (TNF‐α)and interleukin‐6 (IL‐6).^10^, ^11^, ^12^, ^13^, ^14^ The current hypothesis about macrophage polarization is becoming of great importance to researchers around the world. Classically activated (M1 type) macrophages and the alternatively activated (M2 type) macrophages are polarised under different stimulating conditions.^15^, ^16^, ^17^, ^18^, ^19^ M1‐type macrophages have strong phagocytic activity and also release pro‐inflammatory factors, inducible nitric oxide synthase (iNOS).^18^, ^19^, ^20^, ^21^ In contrast, M2‐type macrophages exhibit anti‐inflammatory effects, which produces anti‐inflammatory factors such as IL‐10 and arginase‐1 (Arg‐1).^21^, ^22^, ^23^, ^24^ Ti wear particles promote the release of pro‐inflammatory factors by macrophages.^25^ When Ti particles are produced, the macrophages aggregate and phagocytose Ti particles, as part of the body's autoimmune defence. Therefore, we questioned whether Ti particles had an effect on the polarisation of macrophages before the production of osteoclasts.

Quercetin is a flavonoid with a variety of biological effects, such as inhibition of tumour cell proliferation and migration by anti‐cyclooxygenase‐1; protection of cardiovascular effects; and regulation of the immune system.^26^, ^27^ The inhibition of RANKL‐mediated osteoclast differentiation has been studied,^28^ but to our knowledge, there are no studies of Ti particle‐mediated osteoclast differentiation by the addition of quercetin; indeed, the effects of Ti particle‐mediated macrophages have not been studied in the past. The purpose of this study was to: (a) investigate how the quercetin affects titanium‐mediated osteoclast differentiation, (b) determine the effect of Ti particles on macrophage differentiation in the early stage and (c) explore the mechanism of the quercetin regulation of macrophage polarisation.

## 2 MATERIALS AND METHODS

### 2.1 Reagents

α‐Minimum essential medium (α‐MEM) and foetal bovine serum (FBS) were obtained from Gibco; Thermo Fisher Scientific, Inc. Soluble recombinant mouse macrophage‐colony stimulating factor (M‐CSF) and recombinant mouse RANKL were obtained from R&D Systems, Inc. Tartrate‐resistant acid phosphatase (TRAP) was obtained from Sigma‐Aldrich and Merck KGaA. High purity Ti particles (average diameter. 1‐5 µm) were obtained from Johnson Matthey (cat. no., 00681). The antibodies (GAPDH, NFkB, C‐FOS, NFATc1, p‐p38,) were purchased from Cell Signaling Technology, Inc. Enzyme‐linked immunosorbent assay (ELISA) kits (IL‐6, IL‐1β, TNF‐α, IL‐10, Arg‐1, iNOS) were purchased from R&D Systems, Inc Flow cytometry anti‐mouse CD16/32‐PE (cat. no.553145) and anti‐mouse CD206‐Alexa 647 (cat. no.565250) were purchased from BioLegend Inc The p38α/β MAPK inhibitor (SB202190) was purchased from Selleck.

### 2.2 Preparation of Ti particles

Ti particles were prepared as described previously.^2^, ^3^ The Ti particles were soaked in 75% (v/v) ethanol for 48 hours to remove endotoxins. Next, we used a chromogenic end‐point TAL with Diazo coupling kit (Xiamen Houshiji) to detect endotoxins. When the endotoxin unit (EU) concentration is <0.1 EU/mL, Ti particles could be used for experiments. For the co‐culture, 0.1 mg/mL Ti particles were used. This concentration was similar to that of the wear particles around the prosthesis, as reported previously.^29^, ^30^

### 2.3 Animal experiments

Twenty‐four healthy female C57BL/6 mice (body weight: 16‐18 g) were obtained from the Animal Center Research Committee of the Shanghai Ninth People's Hospital (Shanghai, China). All animal procedures were approved by the animal hospital affiliated to Shanghai Jiao Tong University. A mouse calvarial osteolysis model was used to observe Ti particle‐mediated osteolysis and pro‐inflammatory factor release in vivo. The mice were assigned randomly to one of four groups: control, Ti, low (2 mg/kg/d) concentrations and high (5 mg/kg/d) concentrations of the quercetin for 14‐day. The mouse was killed after day 14. Paraformaldehyde was used to soak the samples. Next, samples were scanned by micro‐CT (Skyscan 1072; Skyscan). The bone volume/tissue volume (BV/TV) and bone mineral density (BMD) were analysed.^4^

### 2.4 H&E and TRAP staining

Decalcification solution (10% ethylenediaminetetraacetic acid (EDTA)) was applied for 7 days, and then, samples embedded in paraffin. Histological sections (4 μm) were prepared for H&E and TRAP staining. Finally, the sample was observed in a light microscopy (magnification, 10×). Images were analysed using Image‐Pro Plus 6.0 (Media Cybernetics, Inc).

### 2.5 Immunohistochemistry and immunofluorescence

The histological sections were prepared for immunohistochemistry and immunofluorescence assay. Antibody IL‐1β (mouse, 1:200), antibody TNF‐α (mouse, 1:200) and antibody IL‐6 (mouse, 1:200) were used for immunohistochemistry. The antibodies CD206 (mouse, 1:200), iNOS (mouse, 1:200), OPG (mouse, 1:200) and RANKL (mouse, 1:200) were used for immunofluorescence assay. All antibodies were purchased from Cell Signaling Technology. In addition, periprosthetic interface tissues were sampled at the time of revision surgery. This study was approved by the local Ethics Committee. The samples were embedded in paraffin. The histological sections were prepared for immunohistochemistry and immunofluorescence assay. The antibodies IL‐1β (human, 1:200), TNF‐α (human, 1:200) and IL‐6 (human, 1:200) were used for immunohistochemistry. The antibodies CD206 (human, 1:200) and iNOS (human, 1:200) were used for immunofluorescence assay.

### 2.6 CCK‐8 assay

The effect of different concentrations of the quercetin on RAW cell proliferation was observed by the Cell Counting Kit‐8 Assay (CCK‐8, Dojindo). Cells（1.0 × 10^4^/well）were plated onto 96‐well plates. After 48 hours, the culture solution (containing 10 μL CCK‐8 solution and 90 μL normal medium) was added to each well at 37°C for 2 hours. Finally, the absorbance microplate reader (Bio‐Tek) was used to measure the optical density (OD) at a wavelength of 450 nm.^3^

### 2.7 TRAP staining, F‐actin ring formation assay and bone pits resorption assay

Bone marrow macrophages (BMMs, 1 × 10^4^ cells/well) were seeded in 96‐well plates for TRAP staining and F‐actin staining. In addition, sterile bone pieces were placed on a 96‐well plate. BMMs (1 × 10^4^ cells/well) were seeded on bone piece for bone pits resorption assay. The culture conditions of BMMs were medium containing M‐CSF (30 ng/mL) and RANKL (50 ng/mL). For these three experiments, the cells were divided into four treatment groups: con, Ti (0.1 mg/mL), Ti (0.1 mg/mL) + low (6.3 μmol/L), Ti (0.1 mg/mL) + high (25 μmol/L). Then, the osteoclasts were fixed in 4% PFA for 20 minutes the TRAP staining kit (Sigma‐Aldrich; Merck KGaA) was used to detect the osteoclasts activity, and the cytoskeleton was detected by the rhodamine‐conjugated phalloidin (Cytoskeleton, Inc). The osteoclasts were observed by optical microscopy. If the number of nuclei in a cell was greater than three, the cell was considered to be TRAP positive. An LSM5 confocal microscope (Carl Zeiss AG) was used to observe the F‐actin ring. A Quanta 250 scanning electron microscope (SEM; FEI; Thermo Fisher Scientific, Inc) with a magnification of 10 kV was used to obtain on the bone surface. Image‐Pro Plus 6.0 (Media Cybernetics, Inc) was used to measure the total area of mature osteoclasts and the bone resorption area.

### 2.8 Reverse transcription quantitative polymerase chain reaction (RT‐qPCR)

The samples were divided into four groups as before. Briefly, the RNeasy Mini kit (Qiagen, Inc) was used to collected the total RNA, which was reverse transcribed into cDNA (Takara Bio, Inc). Finally, the SYBR Premix Ex Taq kit (Takara Biotechnology Co., Ltd.) and an ABI 7500 Sequencing Detection System (Applied Biosystems; Thermo Fisher Scientific, Inc) were used to perform qPCR. The following thermocycling conditions were used: 40 cycles of denaturation at 95°C for 5 seconds and amplification at 60°C for 24 seconds. *GAPDH* was used as the reference gene, osteoclast‐related genes were detected including *NFATc1, C‐FOS,* cathepsin‐K (*CK*) and matrix metalloproteinase 9 (*MMP9*), and all the reactions were run in triplicate. The PCR primers were designed as follows:

**Table:** 

GAPDH	F, 5′‐CACCACCATGGAGAAGGCCG‐3′;
GAPDH	R, 5′‐ATGATGTTCTGGGCAGCCCC‐3′;
NFATc1	F, 5′‐CAACGCCCTGACCACCGATAG‐3′;
NFATc1	R, 5′‐GGCTGCCTTCCGTCTCATAGT‐3′;
CK	F, 5′‐CCTCTCTTGGTGTCCATACA‐3′;
CK	R, 5′‐ATCTCTCTGTACCCTCTGCA‐3′;
MMP9	F, 5′‐AGACGACATAGACGGCATCC‐3′;
MMP9	R, 5′‐TGGGACACATAGTGGGAGGT‐3′;
C‐FOS	F, ACGTGGAGCTGAAGGCAGAAC‐3′;
C‐FOS	R, AGCCACTGGGCCTAGATGATG‐3′;
OPG	F,5′‐CGAGCGCAGATGGATCCTAA‐3′;
OPG	R, 5′‐CCACATCCAACCATGAGCCT‐3′;
RANKL	F, 5′‐CCCATCGGGTTCCCATAAAGT‐3′;
RANKL	R, 5′‐CGACCAGTTTTTCGTGCTCC‐3′;

### 2.9 Small interfering p38α‐RNA and p38β‐RNA

The siRNA specific for p38α, p38β and scrambled siRNA were purchased from RiboBio Company (RiboBio). The RNAiMAX (Thermo Fisher Scientific) and siRNA were used to transfect into RAW cells according to the manufacturer's protocol.

### 2.10 Western blot analysis

The protein expression of NFATc1, C‐FOS and Ikb was measured with and without quercetin treatment in a conditioned medium (M‐CSF, 30 ng/mL; RANKL, 50 ng/mL). In addition, the protein expression of p‐p38, Arg‐1 and iNOS were measured with and without the quercetin, SB202190 (p38α/β MAPK inhibitor), small interfering p38α‐RNA and small interfering p38β‐RNA treatment. The radioimmunoprecipitation assay (RIPA) lysis buffer (cat. no. C500005; Sangon Biotech Co., Ltd.) containing 1 µmol/L protease inhibitor was added to plates for 15 minutes and centrifuged (4°C) at 12 000 *g* for 10 minutes. The bicinchoninic acid assay (BCA) was used to measure the total protein concentration. Equal amounts of the protein lysates were separated via SDS‐PAGE (10% gel), and gels were transferred to polyvinylidene difluoride membranes (PVDF), blocked for 1 hour with 5% (w/v) milk and incubated at 37°C with primary antibodies against GAPDH (cat. no. #8884; 1:1,000; Cell Signaling Technology, Inc), C‐FOS (cat. no. #2250; 1:1,000; Cell Signaling Technology, Inc), NFATc1 (cat. no. #8032; 1:1,000; Cell Signaling Technology, Inc) ikb and p‐ikb(cat. no. #8032; 1:1,000; Cell Signaling Technology, Inc), p38 and p‐p38 (cat. no. #8032; 1:1,000; Cell Signaling Technology, Inc), Arg‐1 and iNOS(cat. no. #13120; 1:1,000; Cell Signaling Technology, Inc) overnight. The horseradish peroxidase‐conjugated secondary antibodies (cat. no. #7074; 1:5,000; Cell Signaling Technology, Inc) reactivity was detected by the Odyssey infrared imaging system (LI‐COR Biosciences).

### 2.11 Enzyme‐linked immunosorbent assay (ELISA)

After 3 days of culture, the concentrations of IL‐1β, IL‐6, IL‐10, TNF‐α, Arg‐1 and iNOS were quantified by using appropriate ELISA kit (R&D Systems) in accordance with the manufacturer's instructions.

### 2.12 Flow cytometry

Raw cells were seeded in 6‐well plates at a density of 4 × 10^5^. By using flow cytometry, the macrophage subpopulation markers CD16/32 (M1) and CD206 (M2) were used to assess different phenotypes. After each group of cells was cultured for 24 hours under different conditions, the cells were trypsinised for flow cytometry analysis. The Mouse CD16/32 PE and the Mouse CD206 Alexa 647 were incubated separately according to the manufacturer's instructions. Finally, they were analysed on a Guava flow cytometer (Millipore). Data were analysed using guavaSoft 3.1.1 software.

### 2.13 Statistical analysis

The data are expressed as means ± standard deviation. Differences among groups were analysed by one‐way analysis of variance and the post hoc tests with the Student‐Newman‐Keuls post hoc test with SPSS software (version 11.0; SPSS Inc). *P* < .05 was considered to indicate a statistically significant difference.

## 3 RESULTS

### 3.1 Establishment of an animal model and effect on osteoclasts differentiation

**Figure 1:**
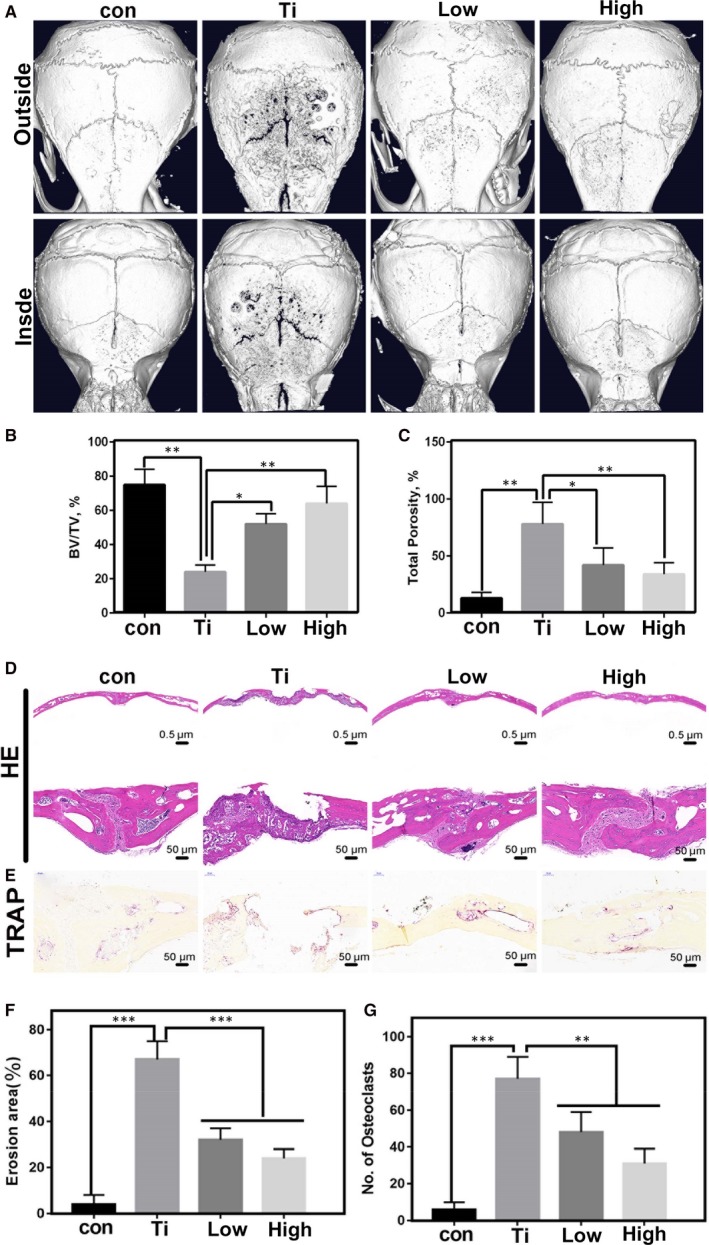
A, The effect of different concentrations of quercetin on titanium particle‐mediated osteolysis by μ‐CT. B and C, Quantitative analysis of the above pictures, including bone volume/tissue volume and total porosity. D and E, It was a histological observation of Ti‐mediated osteolysis and TRAP‐positive osteoclasts. F and G, Quantitative analysis of the tissue sections

μ‐CT images of the Ti particle induced osteolysis model are shown in Figure 1A, including con group, Ti group, Ti+ low group (2 mg/kg/d) and Ti+ high group (5 mg/kg/d). As indicated in Figure 1, the osteolysis of the Ti group was significantly greater than that of the quercetin treatment group. The bone volume/tissue volume in the Ti group was significantly lower than the con group, and the total porosity in the Ti group was higher than that the con group (Figure 1B,C). When the quercetin treatment was administered, osteolysis was significantly ameliorated. From HE and TRAP staining (Figure 1D,E), the phenomenon was similar to that in the μ‐CT image. In addition, we demonstrated that the number of TRAP‐positive cells in the Ti group was significantly higher than that in the con group. But the number of TRAP‐positive cells in the Ti+ low group and Ti+ high group was significantly low (Figure 1F,G).

### 3.2 *The effect on osteoclasts differentiation and function *in vitro

**Figure 2:**
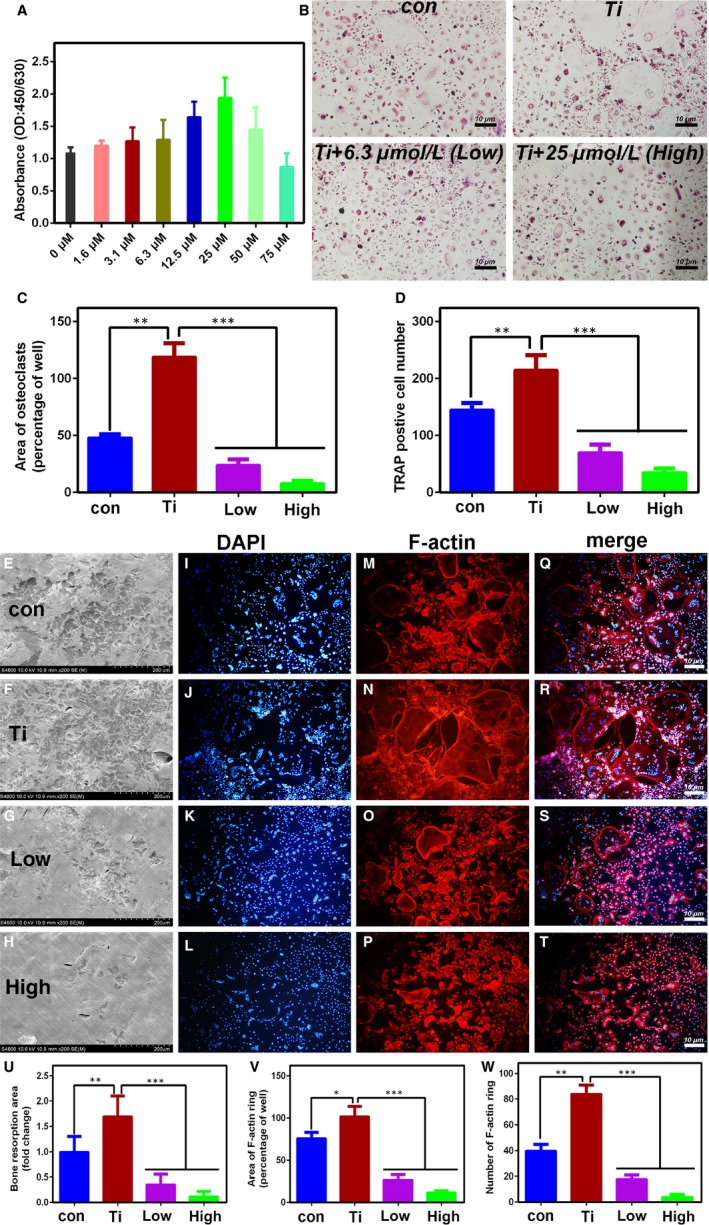
A, Effect of quercetin on cell proliferation. When the concentration reached 50 μmol/L, the cell proliferation was significantly inhibited. B, Effect of quercetin on Ti‐mediated osteoclast differentiation within RANKL. C and D, Quantitative analysis of the above pictures. E‐T, Effect of quercetin on Ti‐mediated osteoclast function. U, Quantitative analysis of the area of bone resorption of the above picture. V and W, Quantitative analysis of the F‐actin of the above picture. The above results demonstrated that Ti promoted the differentiation and maturation of osteoclasts, and this phenomenon could be inhibited by quercetin in vitro

The effect of different concentrations of quercetin on the proliferation of RAW264.7 cells is illustrated in Figure 2A. At a drug concentration of 50 μmol/L, obvious toxic effects on the cells were observed. We also performed CCK‐8 experiments on BMM cells. Similar results to RAW 264.7 cells were obtained from Figure S1. Therefore, we selected low concentration (6.3 μmol/L) and high concentration (25 μmol/L) to verification of the in vitro test. TRAP staining after quercetin treatment and Ti particle treatment are shown in Figure 2B and 2, 2 demonstrated the quantitative analysis of the number of osteoclasts and the area of osteoclasts. We concluded that the quercetin significantly inhibited Ti particle‐mediated osteoclast differentiation. As indicated in Figure 2E‐T, quercetin inhibited the function of pre‐osteoclasts. As shown in Figure 2U‐W, bone pit absorption was significantly inhibited by quercetin, and the formation of F‐actin was also significantly inhibited.

### 3.3 Effects of Res on osteoclast‐related gene, protein and pro‐inflammatory factors

**Figure 3:**
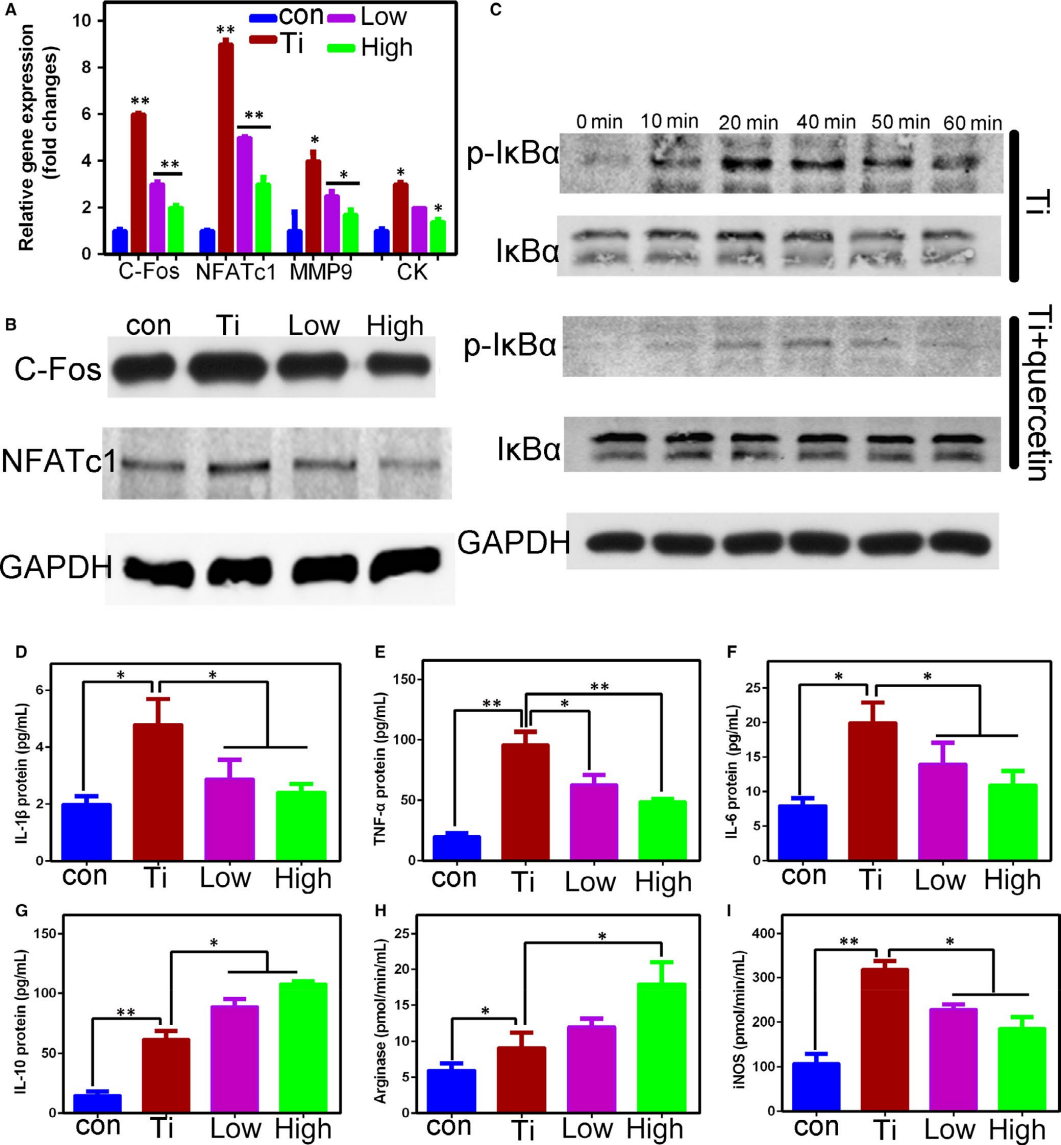
A, Effect of quercetin on Ti‐mediated osteoclast‐associated genes differentiation within RANKL. We concluded that Ti significantly promoted the expression of osteoclast‐associated genes (Nfatc1, C‐fos, Ck, Mmp9) and then be inhibited with the quercetin. B,C, We have demonstrated by Western blotting that Ti promoted osteoclast differentiation by activating NFkB signalling pathway and the related protein (NFATc1, C‐FOS) expression increased significantly. D‐I, We further tested the ELISA and found that the inflammatory factors were significantly increased after the addition of Ti. However, quercetin inhibited the expression of pro‐inflammatory factors (IL‐1β, TNF‐α, IL‐6, iNOS), and on the contrary, quercetin further promoted the expression of anti‐inflammatory factors (IL‐10, Arginase) with addition of Ti. This phenomenon indicated that Ti promoted the differentiation of macrophages into M1 and M2 macrophages. However, quercetin inhibited Ti‐mediated M1 differentiation and promoted the differentiation of M2 macrophages

Next, we further validated the effects of Ti‐mediated osteolysis‐associated genes with the quercetin treatment (Figure 3A). We concluded that the quercetin significantly inhibited genes involved in osteoclast differentiation mediated by Ti particles. As shown in the Figure 3B, quercetin inhibited the C‐Fos and NFATc1 protein. We verified by Western blotting that quercetin inhibited the NFκB signalling pathway (Figure 3C). Next, we also detected inflammatory factors (IL‐6, IL‐1β, TNF‐α, IL‐10, Arg‐1 and iNOS) by using ELISA. We concluded that Ti particles significantly promoted the release of inflammatory factors (Figure 3D‐I). We know that IL‐6, IL‐1β and TNF‐α are pro‐inflammatory factor released by M1 macrophages, whereas IL‐10 is an anti‐inflammatory factor released by M2 macrophages. Therefore, based on ELISA experiments, we verified that Ti particles promoted the secretion of inflammatory factors by M1 macrophages and M2 macrophages. However, quercetin significantly inhibited M1 macrophages mediated by wear particles and promoted M2 macrophages.

### 3.4 Immunofluorescence and immunohistochemistry of osteolytic tissue around the prosthesis in clinical patient

The hip prosthesis of a patient is shown in Figure S2A, with immunofluorescence markers representing M1 macrophages (iNOS) and M2 macrophages (CD206) (Figure S2B). By immunohistochemical analysis, we concluded that M1 macrophages were located around wear particles and promoted the release of inflammatory factors (Figure S2C).

### 3.5 Effects of quercetin on Ti particle‐mediated macrophages polarization

**Figure 4:**
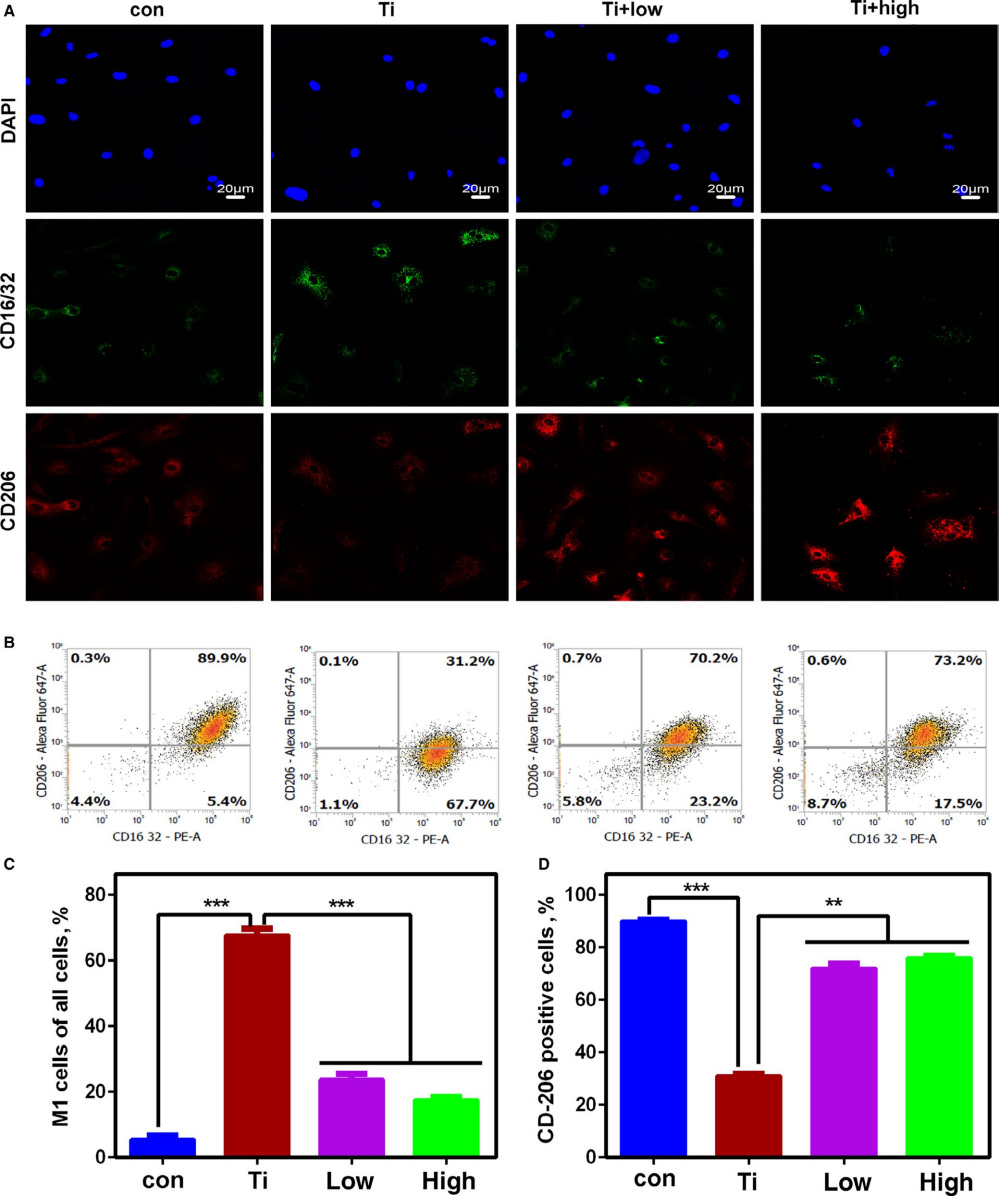
The effect of quercetin on wear particle‐mediated macrophage polarization was further verified in vitro. We confirmed by immunofluorescence (A) that quercetin can inhibited Ti particle‐mediated M1 macrophage polarization and promoted M2 macrophages. We also verified the polarization of this macrophage by flow cytometry (B). The results of flow cytometry were also statistically analysed (C, D). The number of cells collected by flow cytometry is known. If Ti promotes the polarization of M1 macrophages, it is bound to weaken the number of M2 macrophages. Therefore, the number of the CD206 in Figure 5D was significantly reduced

Figure 4A shows the immunofluorescence markers representing M1 macrophages (CD16/32) and M2 macrophages (CD206) in vitro. We revealed that the fluorescence intensity of the CD16/32 in the Ti group was increased. With quercetin treatment, we demonstrated that the fluorescence intensity of the CD206 was increased and that of the CD16/32 was decreased. We further identified the polarization of macrophages mediated by Ti particles by using flow cytometry (Figure 4B). Quantitative analysis of the M1 cells of all cells and the CD206 positive cells are shown in Figure 4C,D. We concluded that Ti particles promoted the polarization of M1 macrophages. Quercetin inhibited the polarization of M1 macrophages and strongly promoted the expression of M2 marker CD206.

### 3.6 Ti particle‐mediated macrophage polarization by p38α/β signalling pathway

**Figure 5:**
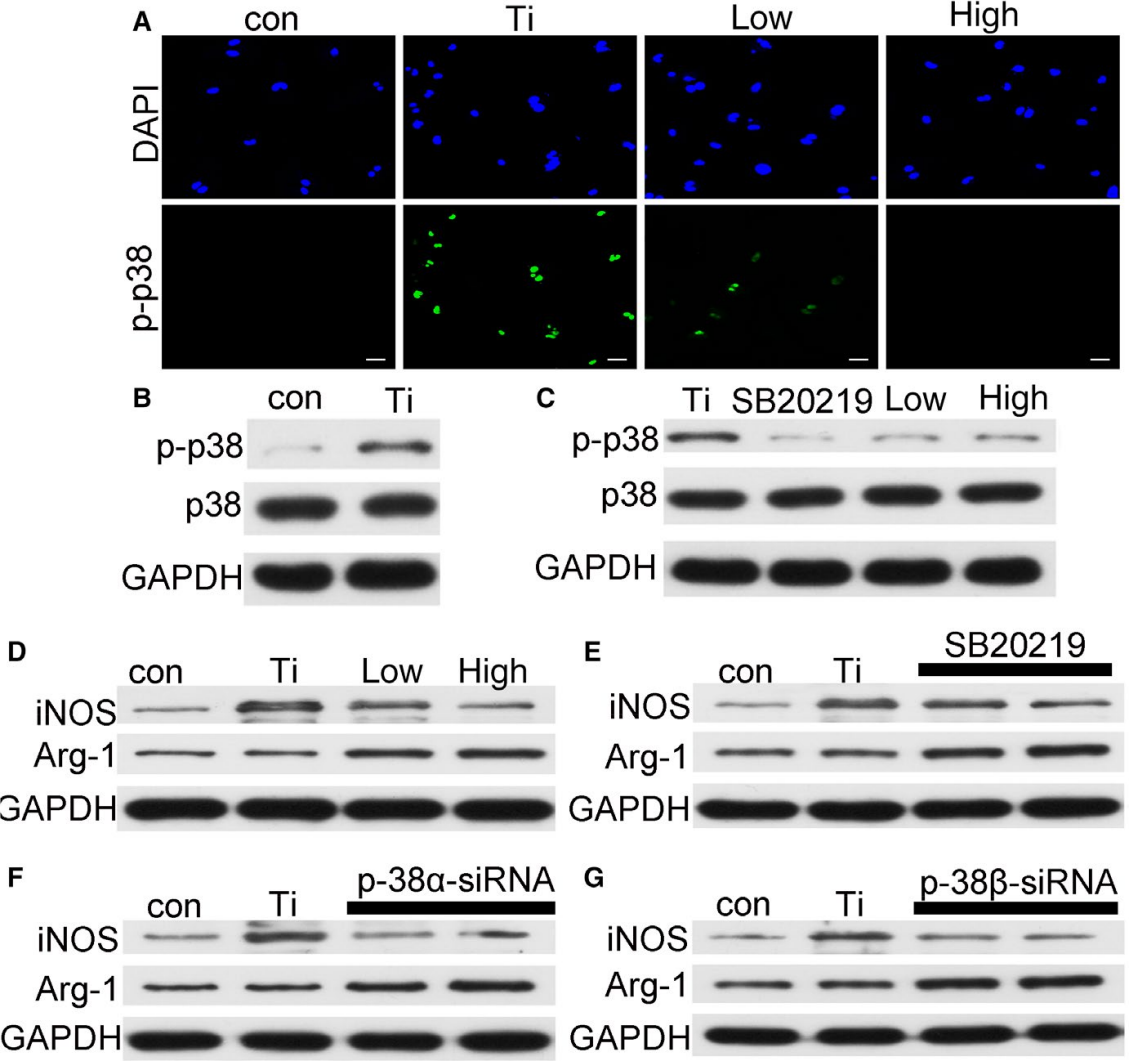
A, The effect of quercetin on p‐p38 by immunofluorescence. B, Ti wear particles activated high expression of p‐p38. C, The inhibitor of p38 and quercetin inhibited the activation of p‐p38 by Ti wear particles. D‐G showed that quercetin, p38 inhibitor, Si‐p38α RNA and Si‐p38β RNA significantly inhibited M1 macrophages from promoting M2 macrophages

As shown in Figure 5A,B. Ti particles promoted the polarization of macrophages by activating the p38 signalling pathway. The phosphorylated p38 protein was significantly attenuated by the addition of quercetin (Figure 5C). It was indicated that quercetin inhibited the polarization of macrophages through the inhibition of phosphorylated p38 (Figure 5D), and when the p38 inhibitor was added (Figure 5E), the Ti‐mediated p38 signalling pathway was significantly inhibited and macrophage polarization was also inhibited. The p38 inhibitor (SB202190) is a p38α/β inhibitor. Therefore, to confirm whether it was an α or a β subunit. We designed small interfering RNA to both p38α and p38β. We found that Ti particle‐mediated macrophage polarization was inhibited after the addition of p38α siRNA and p38β siRNA (Figure 5F,G, respectively).

### 3.7 Effect of macrophage polarisation on histomorphological observation

**Figure 6:**
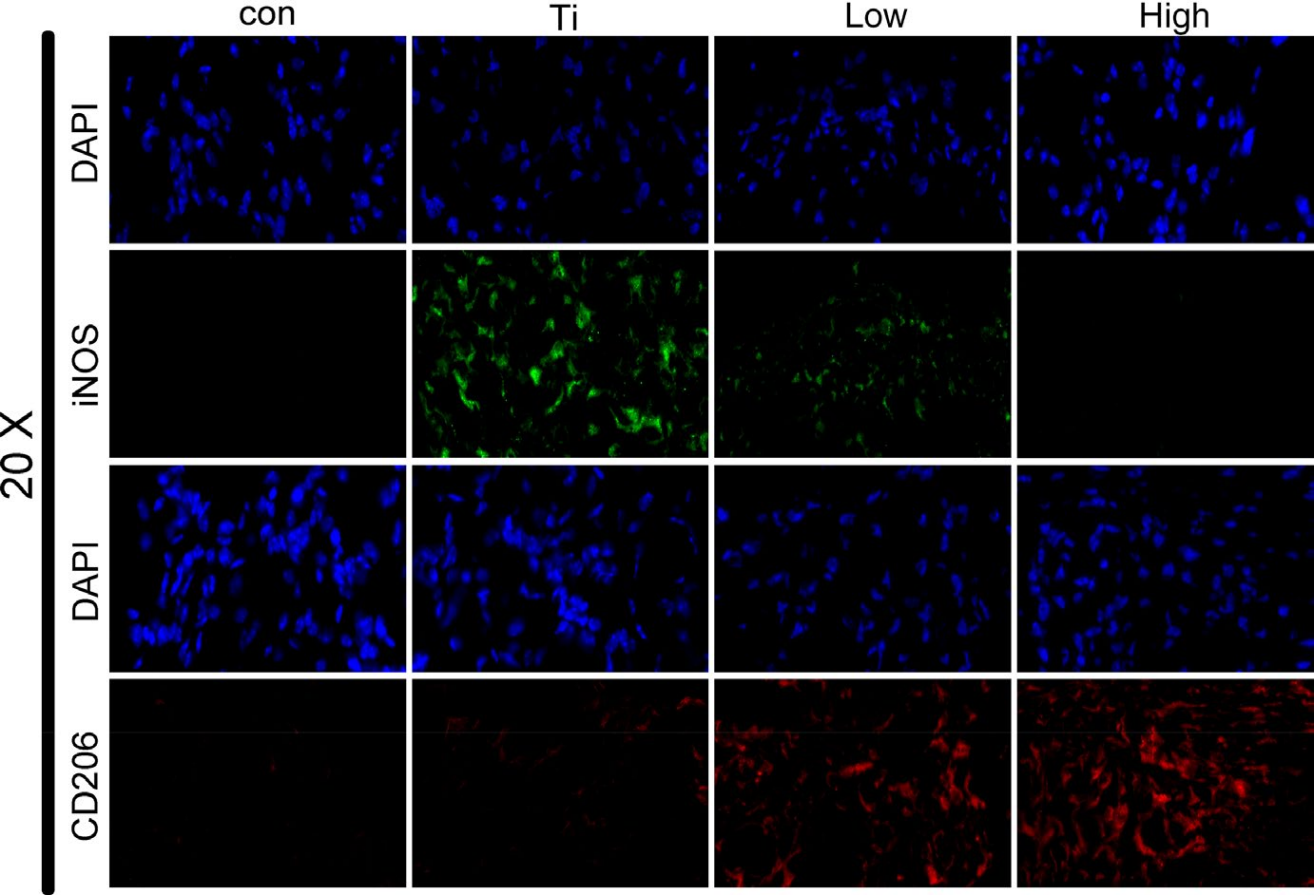
The effect of quercetin on macrophage polarization in vivo. The M1 macrophages in the Ti group were significantly increased by immunofluorescence in a mouse model of osteolysis of the skull. After treatment with quercetin, M2 macrophages increased and quercetin inhibited Ti‐mediated M1 particle‐mediated macrophage polarization

The immunofluorescence of iNOS proteins in tissue sections under different treatment conditions is shown in Figure 6. The expression of iNOS was noted to increase after the addition of Ti and was significantly inhibited by the addition of quercetin. We also performed immunofluorescence analysis of CD206 molecules and found that Ti promoted the increase of M2 macrophages, and that the expression of M2 macrophages increased significantly after treatment with quercetin.

### 3.8 Effect of histomorphological observation on pro‐inflammatory factors, OPG and RANKL

**Figure 7:**
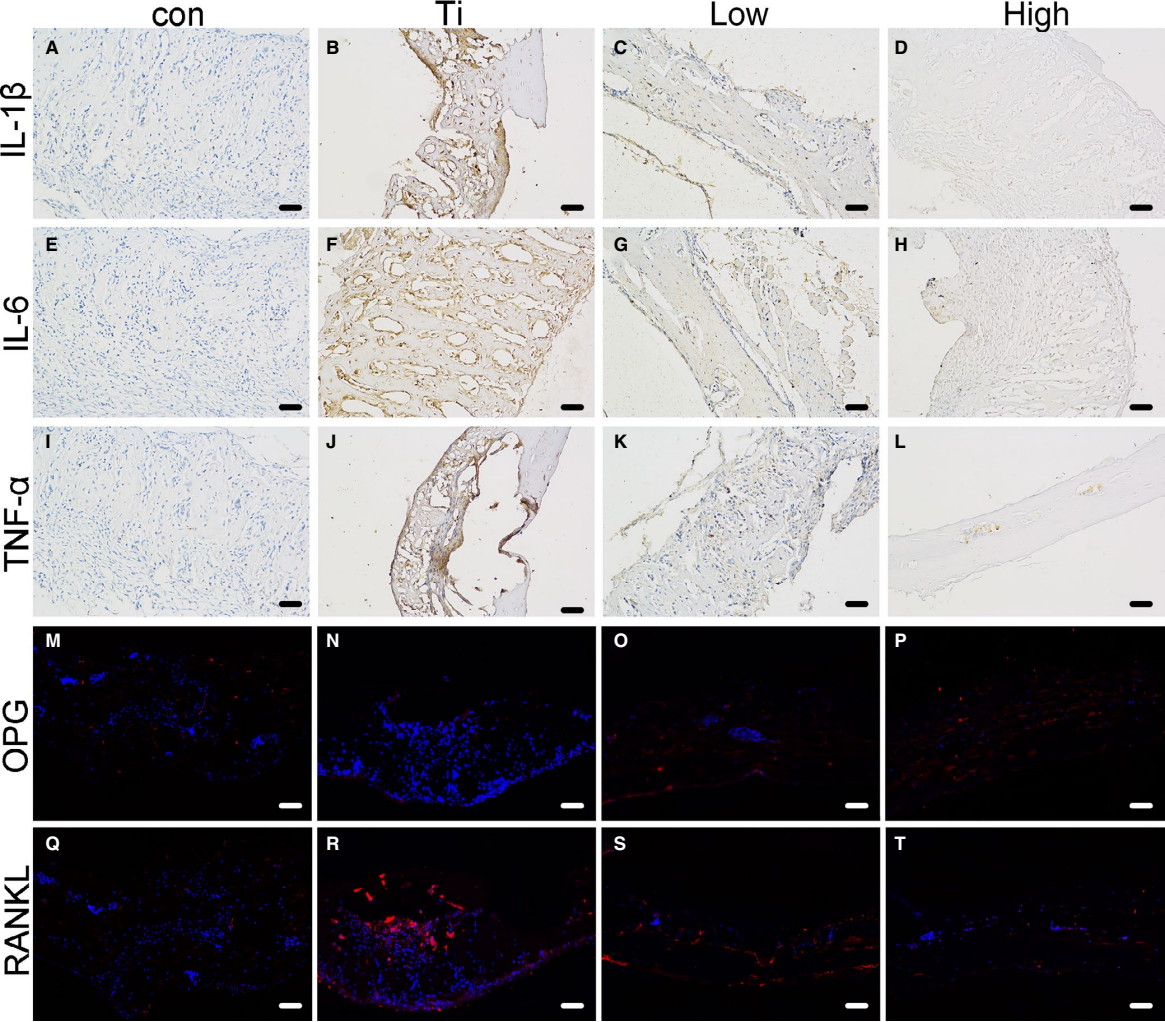
The effect of quercetin on pro‐inflammatory factors (IL‐1β, TNF‐α, IL‐6) by immunohistochemistry (A‐L). We found that Ti significantly promoted the release of pro‐inflammatory factors. The production of the pro‐inflammatory factor was significantly reduced after quercetin treatment. We further made the immunofluorescence of OPG/RANKL. We found that Ti significantly promoted the high expression of the RANKL. However, the RANKL expression was significantly inhibited, but OPG protein expression increased with addition to the quercetin (M‐T)

The above results demonstrated that the quercetin inhibited the macrophage polarization through the p38α/β signalling pathway. Thus, we further performed an immunohistochemistry analysis of the IL‐6, the IL‐1β and the TNF‐α and found that the three proteins were highly expressed in the Ti group. However, upon addition of quercetin, the release of pro‐inflammatory factors was significantly inhibited, and the inhibitory effect occurred in a dose‐dependent manner (Figure 7A‐L). Next, to validate the effect of Ti particles on OPG/RANKL, we performed an immunofluorescence assay of the OPG, RANKL protein (Figure 7M‐T). Compared with the con group, in the Ti group, the expression of OPG was significantly lower and the expression of RANKL was significantly higher, which resulted the imbalance the OPG/RANKL. However, quercetin could relieve the imbalance.

## 4 DISCUSSION

Osteolysis around the prosthesis remains one of the serious complications of total hip arthroplasty. Nowadays, improved prosthetic materials have been designed that inhibit the generation of wear particles.^31^ However, the hip joints in the weight‐bearing position are often subject to biomechanical effects, and it is inevitable that wear particles will be produced after several decades.^32^, ^33^ If wear particles are produced, they stimulate the aggregation of macrophages. Macrophages phagocytose wear particles (Figure S3), releasing a large number of pro‐inflammatory factors (IL‐1β, TNF‐α, IL‐6). These pro‐inflammatory factors are secreted by M1 macrophages. Therefore, we hypothesize that macrophages will transform into M1 macrophages in the presence of wear particles. Moreover, we sampled the osteolytic tissue around the prosthesis, performed immunofluorescence and immunohistochemical analyses and found that most of the macrophages surrounding the prosthesis were M1 macrophages (Figure S2B) that released a large number of pro‐inflammatory factors (Figure S2C) These pro‐inflammatory factors regulate the imbalance of OPG/RANKL.^34^, ^35^, ^36^, ^37^ Numerous studies have shown that the imbalance of OPG/RANKL is an important factor in the production and differentiation of osteoclasts.^38^, ^39^ We also found an imbalance in OPG/RANKL by immunofluorescence of the mouse skull (Figure 7M‐T).

Quercetin (Figure S4) has anti‐inflammatory, antitumour activities and regulates the immune system.^40^, ^41^ It can inhibit the production of osteoclasts through inhibitory effects on the NFkB signalling pathway.^42^ However, the effects of quercetin on Ti particle‐mediated macrophage polarization and Ti particle‐mediated macrophage differentiation into osteoclasts. Therefore, we studied the effects of Ti particles on macrophages and regulated osteoclast differentiation by pro‐inflammatory factors released by macrophages.

First, the animal model used was a classic mouse skull model. The groups are control, Ti particles, Ti particles + a low dose of quercetin and Ti particles + a high dose of quercetin. μ‐CT showed clear osteolysis in the skull of mice in the Ti particle group (Figure 1A). HE and TRAP staining also demonstrated osteolysis and osteoclast formation near Ti particles (Figure 1D,E). However, after treatment with quercetin, osteolysis was significantly inhibited. Second, an in vitro simulation experiment was conducted. We found that the number and area of osteoclasts were significantly inhibited by TRAP staining and that the function of osteoclasts caused by Ti particles was significantly inhibited by F‐actin staining and bone resorption area. Third, we verified, by PCR, that the Ti particle‐mediated osteoclast‐associated genes were inhibited. Ti particles promoted macrophage differentiation into osteoclasts by activating the classical NFkB signalling pathway (Figure 3C). Unexpectedly, we found that the pro‐inflammatory factors secreted by M1 macrophages increased in the presence of Ti particles, M2 macrophages also increased in the Ti group, and quercetin significantly inhibited the increase of M1 macrophages (Figure 3D‐I).

Based on the above experimental results, osteoclasts do not appear immediately when Ti particles are present. Owing to their own immune regulatory system, macrophages firstly gather around Ti particles. Therefore, we simulated this physiological phenomenon again in vitro without the addition of RANKL cytokines. The marker of M1 macrophages increased significantly in the presence of Ti particles (Figure 4A). However, the marker of M1 macrophages decreased significantly in the presence of Ti particles and quercetin. On the contrary, the marker of the M2 macrophages was increased. We have also demonstrated this phenomenon through flow cytometry (Figure 4B). But, we found that the number of CD206 cells in the Ti group was reduced, which may be compared with the total number of cells collected. The number of M1 types has increased, and the number of relative double positive cells has decreased. Therefore, we conclude that Ti particles promote the polarization of macrophages to M1 macrophages, which are pro‐inflammatory macrophages, and consequently secrete a large number of pro‐inflammatory factors (IL‐1β, TNF‐α, IL‐6). This phenomenon has also been confirmed from the histological staining of osteolysis around the prosthesis. Quercetin attenuated Ti‐mediated macrophage polarisation (Figure 6A) reduced the release of pro‐inflammatory factors (Figure 7A‐L). Next, we found that Ti particles promoted the polarization of M1 macrophages by activating the p38 signalling pathway (Figure 5A,B) and the effect could be significantly alleviated by a p38 inhibitor (Figure 5C). However, the inhibitor was targeted to the α/β subunit in the p38 protein.^43^ Next, we validated the specific activation target of Ti particles by small interfering p38α‐RNA and small interfering p38β‐RNA (Figure 5F,G).

Next, we performed histological analysis of the mouse skull through immunohistochemistry and immunofluorescence. We found significantly higher inflammatory factors in the Ti group than in the control group by using immunohistochemistry and that quercetin significantly inhibited the production of pro‐inflammatory factors. In vitro experiments demonstrated that pro‐inflammatory factors are released by Ti‐mediated M1 macrophages. Therefore, we further made immunofluorescence on the tissue sections. We found that Ti‐mediated M1 macrophages increased significantly, whereas M2 macrophages increased in the quercetin‐treated Ti group. It showed that quercetin could regulate the balance between M1/M2. When M1 macrophages increase, they promote the release of pro‐inflammatory factors. Pro‐inflammatory factors further regulate OPG/RANKL imbalance (Figure S5).^44^, ^45^ Then, they could promote the differentiation of macrophages to osteoclasts with RANKL factors, eventually leading to bone resorption effects.

In summary, (a) Ti particles promoted macrophage polarization by activating the p‐38α/β signalling pathway. We validated this pathway by using small interfering siRNA to inhibit of p38α and p38β. (b) Ti particle‐mediated M1 macrophages caused further release of pro‐inflammatory factors (IL‐1β, TNF‐α, IL‐6). (c) Quercetin inhibited macrophage polarization through the p38 signalling pathway and inhibited the release of pro‐inflammatory factors by M1 macrophages in vitro. (d) Ti particles promoted the differentiation of RANKL‐mediated osteoclasts, and the quercetin inhibited osteoclast differentiation via the NFkB signalling pathway in vitro. (e) In vivo, we found that CD16/32(M1) markers increased significantly, CD206(M2) markers were significantly reduced by immunofluorescence and pro‐inflammatory cytokines (IL‐1β, TNF‐α, IL‐6) were significantly increased in the Ti group. (f) In vivo, pro‐inflammatory factors (IL‐1β, TNF‐α, IL‐6) changed the balance of OPG/RANKL, which in turn promoted the differentiation of osteoclasts mediated by RANKL. The osteoclasts promoted osteolysis around Ti particles. (g) Finally, The M1 macrophages and the pro‐inflammatory factors in the tissue around patient's prosthesis are increased.

## CONFLICTS OF INTEREST

There are no conflicts to declare.

## AUTHOR CONTRIBUTIONS

Yu‐Wei Ge, Kai Feng and Xiao‐Liang Liu contributed equally to this study. Zhen‐An Zhu, Hong‐Fang Chen and Yong‐Yun Chang designed the study. Zhen‐Yu Sun and Hao‐Wei Wang analysed the data and drafted the manuscript. Jing‐Wei Zhang, De‐Gang Yu and Yuan‐Qing Mao helped revise the manuscript. All authors reviewed the manuscript and approved the final manuscript. All authors read and approved the final manuscript.

## Data Availability

The data that support the findings of this study are available from the corresponding author upon reasonable request.
