# Supplementary material for: Quercetin inhibits macrophage polarization through the p‐38α/β signalling pathway and regulates OPG/RANKL balance in a mouse skull model
Source: J Cell Mol Med. 2020 Feb 13;24(5):3203–16. doi: 10.1111/jcmm.14995 (PMC7077538; doi:10.1111/jcmm.14995)
Supplement: Supplementary file 1 [file JCMM-24-3203-s001.docx]

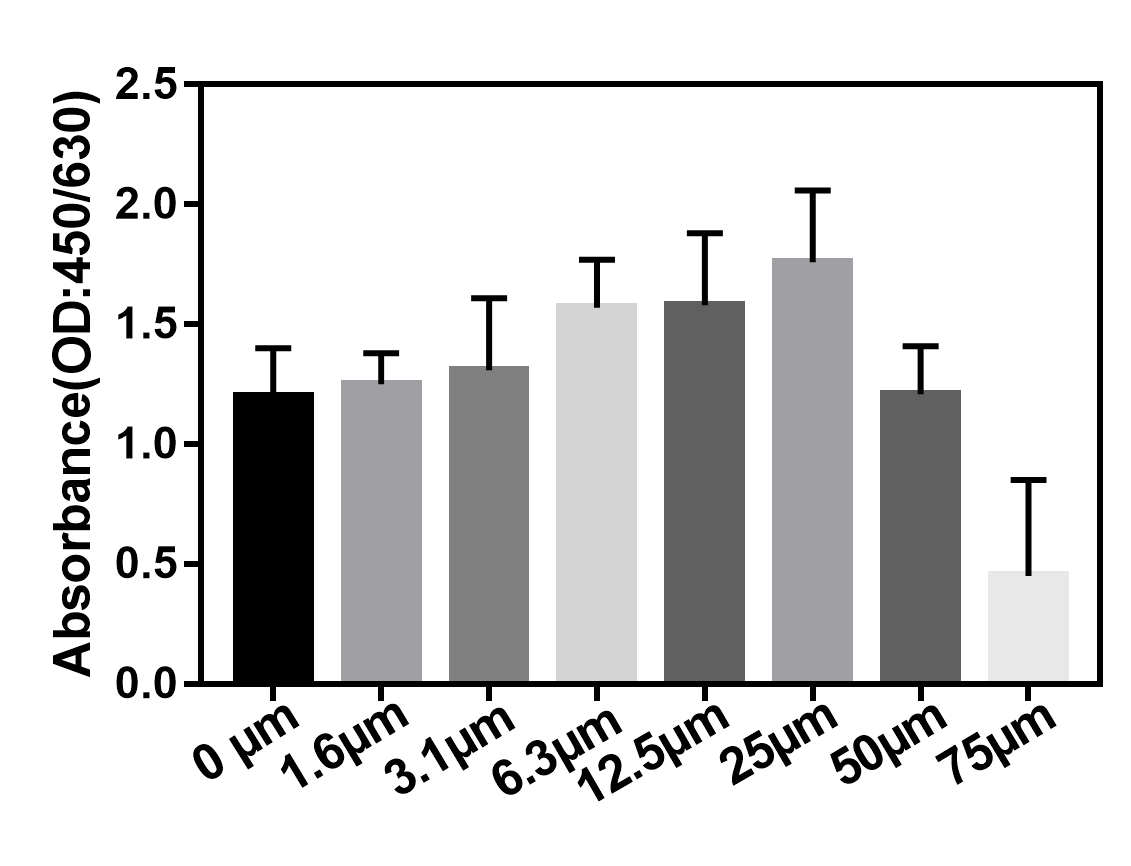


Supplementary Figure. S1 Effect of quercetin on BMM cells proliferation.


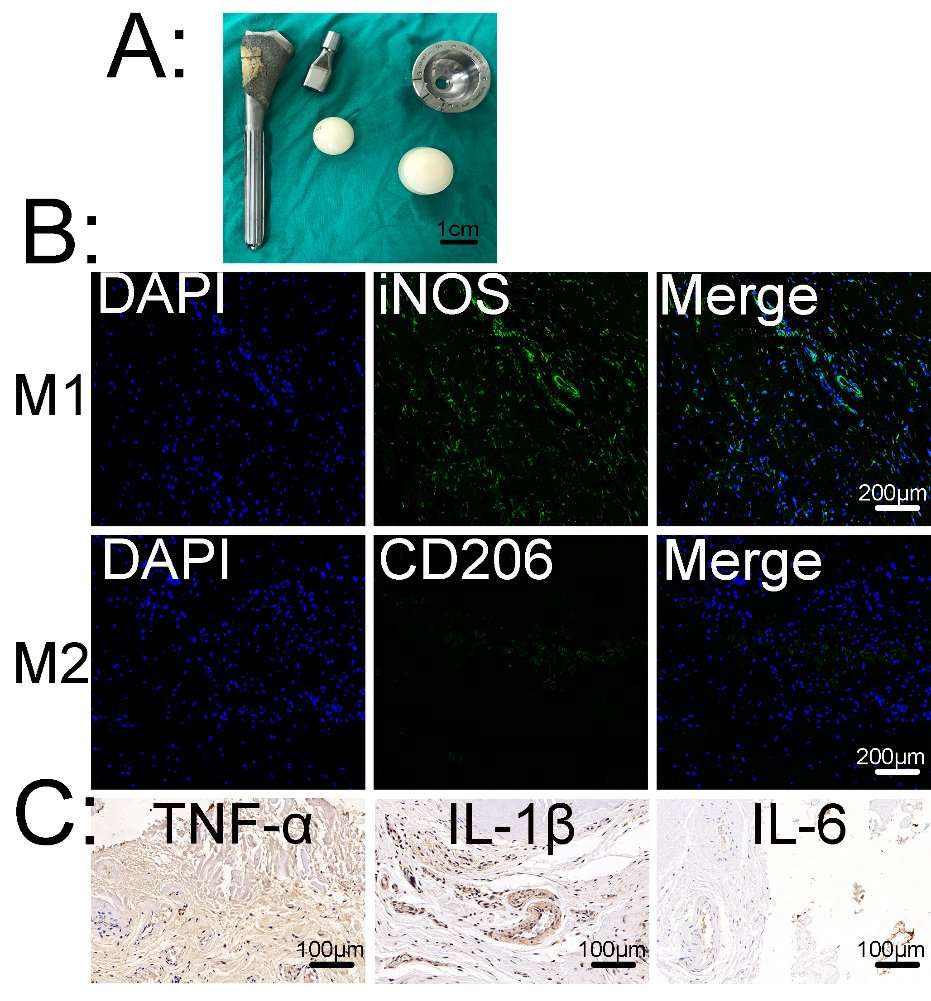


Supplementary Figure. S2. (A) The prosthetic sample of osteolytic patients. (B) Immunofluorescence of bone tissue around the prosthesis. The iNOS represents M1 type macrophages and the CD206 represents M2 type macrophages. We found that both M1 macrophages and M2 macrophages were expressed, but most of the cells surrounding the prosthesis presented M1 macrophages. (C) We further carried out immunohistochemistry of pro-inflammatory factors(IL-1β，TNF-α，IL-6) in bone tissue.


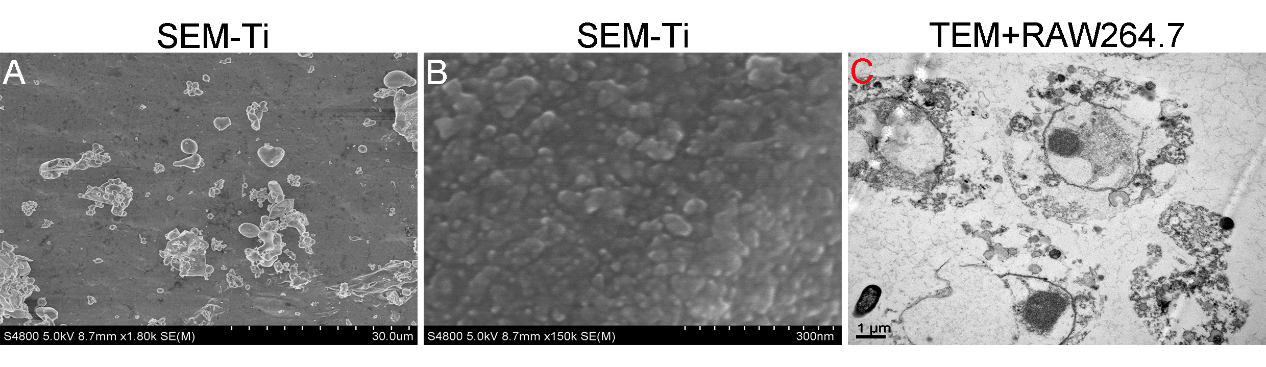


Supplementary Figure. S3. A is a low magnification scanning electron micrograph of titanium wear particles. B is a high magnification scanning electron micrograph of titanium wear particles. C is a transmission electron micrograph of RAW cells phagocytized titanium particles, and it showed that small particles of titanium were engulfed by RAW cells.


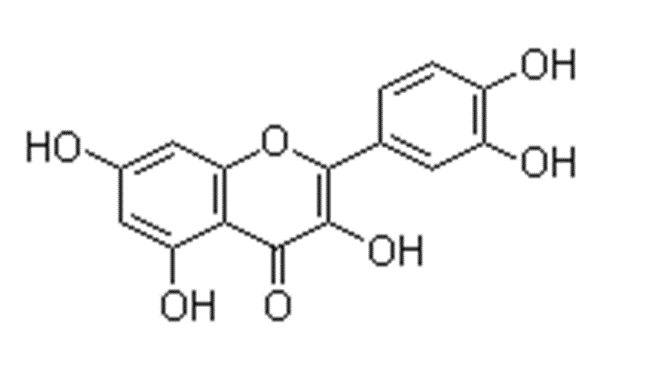


Supplementary Figure. S4. Chemical structure of quercetin


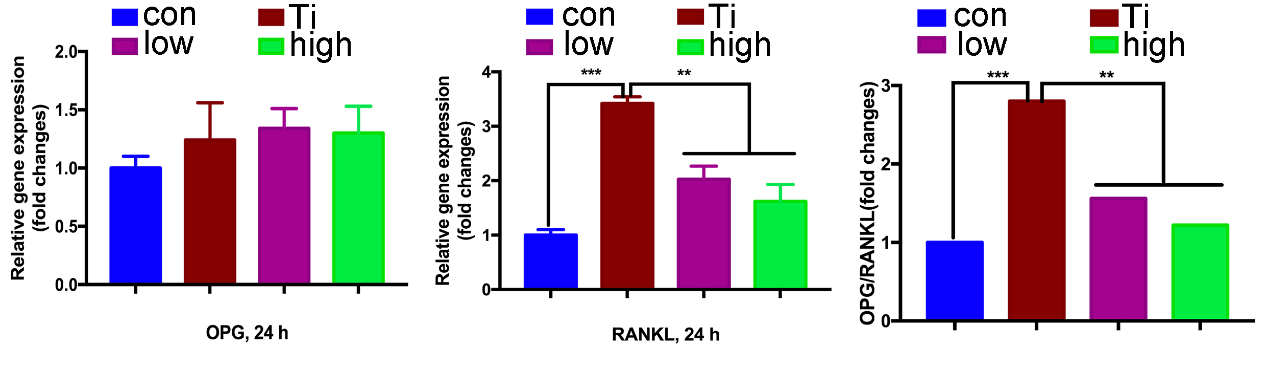


Supplementary Figure. S5. The effect of the culture medium of raw cells on the osteoblast OPG and RANKL genes under different treatment factors. The effect on OPG gene was not statistically significant within 24 hours. On the contrary, it could change the gene expression of RANKL, and then making OPG/RANKL unbalanced.
